# Physical and Chemical Relationships in Accelerated Carbonation Conditions of Alkali-Activated Cement Based on Type of Binder and Alkali Activator

**DOI:** 10.3390/polym13040671

**Published:** 2021-02-23

**Authors:** Yuto Yamazaki, Jihoon Kim, Keisuke Kadoya, Yukio Hama

**Affiliations:** 1 Department of Civil Engineering and Architecture, Muroran Institute of Technology, Hokkaido 050-8585, Japan; 19041087@mmm.muroran-it.ac.jp (Y.Y.); hama@mmm.muroran-it.ac.jp (Y.H.); 2 Department of Construction, Okumura Corporation Sapporo Branch, Hokkaido 060-0004, Japan; keisuke.kadoya@okumuragumi.jp

**Keywords:** alkali-activated cements, alkali activator, compressive strength, XRD, TG-DTG, ^29^Si MAS NMR, C–(N)–A–S–H, N–A–S–H

## Abstract

Alkali-activated cements prepared from aluminosilicate powders, such as blast furnace slag and fly ash, are rapidly attracting attention as alternatives to cement because they can significantly reduce CO_2_ emissions compared to conventional cement concrete. In this study, we investigated the relationship between the physical and chemical changes by accelerated carbonation conditions of alkali-activated cements. Alkali-activated cements were prepared from binders composed of blast furnace slag and fly ash as well as alkali activators sodium silicate and sodium hydroxide. Physical changes were analyzed from compressive strength, pH, and neutralization depth, and chemical changes were analyzed from XRD, TG-DTG, and ^29^Si MAS NMR. The C–(N)–A–S–H structure is noted to change via carbonation, and the compressive strength is observed to decrease. However, in the case of Na-rich specimens, the compressive strength does not decrease by accelerated carbonation. This work is expected to contribute to the field of alkali-activated cements in the future.

## 1. Introduction

**Figure 1:**
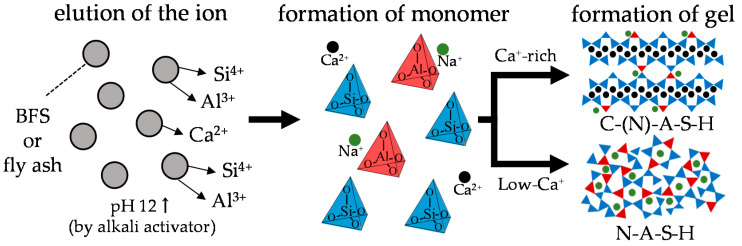
Reaction mechanisms of alkali-activated slag and fly ash.

The production of ordinary Portland cement (OPC), which is the main material in conventional concrete structures, has increased rapidly in recent years. However, the production of OPC is responsible for releasing a large amount of CO_2_ into the atmosphere, and these emissions can be assessed from the viewpoint of two aspects. The first aspect is the chemical reaction involved in the production of the main component of cement, i.e., clinker, as carbonates (largely CaCO_3_, found in limestone) that are decomposed into oxides (largely lime, CaO) and CO_2_ (CaCO_3_ → CaO + CO_2_↑) by the application of heat. The second aspect involves consumption of large amounts of fossil fuels that are burned at about 1400 °C. Owing to these two causes, approximately 0.75–0.85 tons of CO_2_ is typically emitted to produce 1 ton of Portland cement. The total CO_2_ emissions from the cement industry have increased rapidly in recent years, accounting for about 4% [1] of the total CO_2_ emissions in Japan and 8% [2] globally, and constitute the third largest source of anthropogenic CO_2_ emissions. The demand for reducing CO_2_ emissions is thus increasing as a measure for mitigating environmental problems, such as global warming; hence, materials that can sufficiently replace cement are required. In recent years, alkali-activated cements (AAC_S_) have been studied as building materials to replace cement. AAC_S_ are generally composed of aluminosilicate powders, such as blast furnace slag (BFS), fly ash (FA), and metakaolin (MK), along with alkali activators such as sodium silicate (Na_2_O·SiO_2_·xH_2_O; water glass) and sodium hydroxide (NaOH). OPC hardens via hydration reaction with water, but in the case of the AAC_S_, Si^4+^, Al^3+^, and Ca^2+^ contained in the binder elute and react with the alkali activators (Figure 1). It has been reported that AAC_S_ have a CO_2_ reduction effect of about 80% compared to OPC because the AAC_S_ do not contain OPC [3]. In addition, AAC_S_ have excellent strength development [4], better acid resistance [5,6,7], and better heat resistance [8,9,10] than normal cement concrete. 

Aluminosilicate powders (or alkali-activated cements) such as BFS, FA, and MK used in AACs have different chemical composition ratios of Ca, Si, and Al, compared to OPC. In addition, there is a supply of alkali metals (Na, K) and Si from the alkali activator used. Therefore, it is understood that hydrates different from OPC are produced [11,12,13]. In the case of OPC, calcium silicate hydrate (C–S–H) is formed as the main hydration product. BFS-based AACs produce calcium aluminosilicate hydrate (C–A–S–H, C–(N)–A–S–H) because the BFS has a higher Al^3+^ content than OPC; C–(N)–A–S–H has a more polymerized nanostructure than C–S–H. However, FA-based AACs with low calcium form sodium aluminosilicate hydrate (N–A–S–H, K–A–S–H) containing large amounts of alkali metal ions such as Na^+^ and K^+^ as well as Si^4+^; N–A–S–H has a more polymerized three–dimensional structure than C–(N)–A–S–H. Therefore, these AAC_S_ have different physical and chemical properties from conventional OPC concrete. Moreover, the properties vary depending on the chemical compositions of the binder and concentrations of the alkali activators. 

Therefore, the chemical compositions and physical properties of the alkali activators and binders can significantly change the properties of the pore structures and hydration products of AACs [14,15,16,17]. In recent years, there have been several studies on AACs as well as studies for their practical application. Most of the research to date has focused on specific properties of the AACs, such as their compressive strength [18]. In addition, due to these differences in composition, AACs exhibit different tendencies in long-term durability, such as carbonation, compared to OPC concrete. Therefore, it is necessary to evaluate the chemical as well as physical properties. In the case of carbonation of OPC concrete, CaCO_3_ products such as calcite and vaterite are produced from Ca(OH)_2_, C–S–H, and monosulfate (AFm) by carbonation. The pH of the concrete is lowered by consumption of Ca(OH)_2_, and its compressive strength is increased by densification of the pore structure owing to the formation of CaCO_3_ [19]. However, in the case of carbonation of BFS-based AACs, the reaction progresses faster than in OPC concrete because the Na^+^ contained in the alkali activator promotes elution of Ca^2+^ contained in C–(N)–A–S–H [20,21,22].

A. Bernal reports in detail the changes in the gel nanostructure due to carbonation of AACs through experiments using paste specimens based on BFS and FA [23]. In the experiment by CO_2_ concentration, he describes the inconsistency between the mechanism of carbonation in the laboratory’s high CO_2_ environment and in the real environment. In such high concentration CO_2_ conditions, in the case of carbonation of BFS-based AACs, carbonation may reduce the compressive strength depending on the reaction conditions [22,24,25,26]. F. Puertas et al. [22] showed that when using sodium hydroxide as the alkali activator, the compressive strength increased by carbonation; however, when using sodium silicate, the compressive strength decreased by carbonation. Thus, we considered that the physical properties after carbonation would differ depending on the alkali activator used. Moreover, M.S.H. Khan et al. [27] showed that in the carbonation of low-calcium FA-based AACs, the pH was lowered by carbonation, as in the case of OPC. However, M. Nedeljkovic et al. [26] showed that the carbonation does not change the compressive strength in the case of FA-based AACs. Therefore, we considered that the physical properties after carbonation also differed depending on the binder used. Summarizing the results of these existing literature, the composition of each binder and the supply of alkali activators exhibit different physical and chemical changes in carbonation under high CO_2_ conditions. In addition, CO_2_ penetration and change in pH from the specimen matrix surface may also have an effect on the change in physical properties. For studies on materials used in real structures such as concrete and cement, it is very important to assume real environmental conditions. However, many studies are being conducted under accelerated deterioration conditions in the laboratory, and it is also important to predict the behavior under real environmental conditions from accurate evaluation at the laboratory level. 

Therefore, in this study, we consider the mechanism of carbonation at the laboratory level, i.e., in accelerated carbonation environments, in high CO_2_ environments. Based on the existing literature, we aim to evaluate the physical and chemical changes and their relationship to the carbonation mechanism according to various binders and alkali activators. Mortar and paste specimens were prepared using the BFS and FA and BFS:FA mixed in a 1:1 ratio substances as the binders, and sodium hydroxide (SH) and sodium silicate (SS) as the alkali activators. Changes in the compressive strengths, neutralization depths, pH values, and hydration products by XRD and ^29^Si MAS NMR before and after carbonation were confirmed.

## 2. Materials and Methods 

### 2.1. Materials and Procedures

The binder and alkali activators were set based on previous study [23], BFS (B100), FA (F100) and BFS:FA mixed in a 1:1 ratio (B50F50) was used. Table 1 shows the physical properties and chemical compositions of the BFS and FA used. Two types of alkali activators were also used, namely sodium silicate (SS, Na_2_O = 18.0 wt.%, SiO_2_ = 36.5 wt.%, H_2_O = 45.5 wt.%) solution diluted about 2.1 times and 10 mol/L sodium hydroxide (SH1). The amount of activator added was determined from the ratio of Na_2_O/binder. In addition, SH2 was set to have the same Na^+^ concentration as SS for comparison according to Na^+^ concentration. For our experiments, all the specimens were prepared at a water–cement ratio (W/B) of 0.4. Table 2 and Table 3 show the mix preparations of the mortar and paste specimens. In this study, mortar specimens were used to measure the compressive strengths, neutralization depths, and the paste specimens were used for the XRD, thermogravimetric/differential thermal gravimetric (TG/DTG), and ^29^Si MAS NMR analyses (Table 4). The fine-aggregate (density: 2.68 g/cm^3^, water absorption ratio: 2.17 wt.%) to binder ratio of mortar was set to 2:1, and the size of each mortar specimen was 40 × 40 × 160 mm^3^ (neutralization depths) and φ50 mm × height 100 mm (compressive strengths). The paste specimens were crushed after curing and used for the measurements.

Immediately after the casting, all the specimens were subjected to sealing and curing at 20 °C for 2 h. Thereafter, high-temperature sealed curing was performed at 60 °C for 6 h. Subsequently, sealed curing was performed again under 20 °C, and the molds were removed 24 h after mixing. Finally, all specimens were cured in air condition at 20 °C and 60% relative humidity (RH) for up to 8 weeks. The accelerated carbonation tests were carried out at 4 weeks and 8 weeks under the conditions of CO_2_ concentration of 5%, temperature of 20 °C, and RH of 60% using the cured specimens.

### 2.2. Compressive Strength

The compressive strength of the mortar was measured (Industrial Series DX600, Instron Japan, Kawasaki, Japan) in accordance with JIS-A-1108 [28] at each age (8, 12, and 16 weeks). The load was uniformly applied to such a degree that no impact was applied. The loading speed was set to 0.6 ± 0.4 N/mm^2^ per second. The compressive strength was measured five times per each sample level, and the results of compressive strength are represented by three average values excluding minimum and maximum values.

### 2.3. X-ray Diffraction

XRD was performed to identify the changes of crystalline phase. A Rigaku-SmartLab powder diffractometer (Tokyo, Japan) was used for measurements. The XRD conditions were as follows: Cu-Kα radiation resource; 40kV; 30mA; scan range, 3–70°/2θ; scan speed, 2°/min; step width, 0.02°/step.

### 2.4. Thermogravimetric/Differential Thermal Gravimetry

TG/DTG (STA 7200, HITACHI, Tokyo, Japan) was performed on samples to examine the thermal decomposition, in a nitrogen atmosphere, from 20 to 1000 °C at a heating rate of 20 °C/min. All measurements were performed with 10 mg of powder.

### 2.5. Solid-State Nuclear Magnetic Resonance

^29^Si MAS NMR spectra were collected at 99.4 MHz on JEOL ECA-500 (magnetic field 11.75T, Tokyo, Japan) using a 3.2 mm φ probe. The ^29^Si NMR experiments employed a spinning speed at 10 kHz, a pulse width of 3.6 μs, a relaxation delay of 15 s and total 2500 scans. Analysis of the solid-state NMR spectra was performed on a JEOL Delta NMR processing and control software.

### 2.6. pH Measurement and Phenolphthalein Method

We used a 40 × 40 × 160 mm^3^ mortar specimen and sprayed a 1% solution of phenolphthalein on a 40 × 40 mm^2^ split section to observe the color change. The pH was measured with a solution of 20 mL of pure water and 1 g of powder collected from two locations on the surface side (5 mm from the surface) and the center side (20 mm square in the center) of the 40 × 40 mm^2^ split section. 

## 3. Results

### 3.1. Compressive Strength

**Figure 2:**
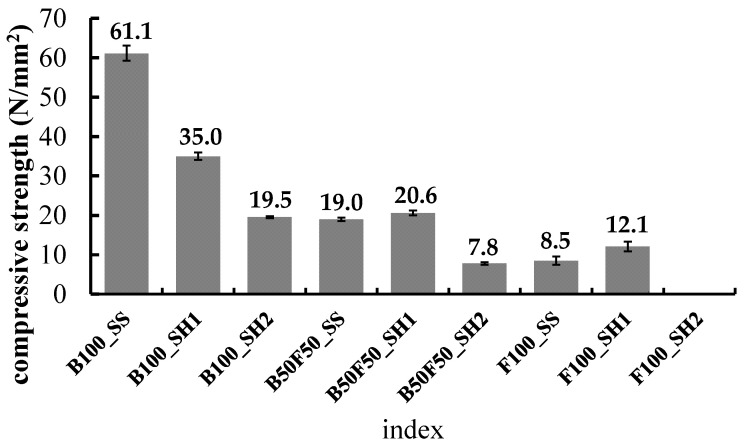
Compressive strength of each specimen at 8 weeks.

**Figure 3:**
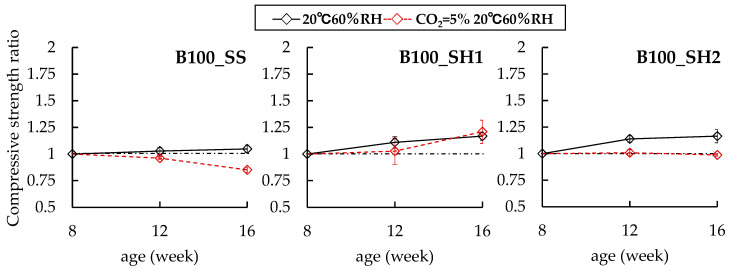
Changes in compressive strength ratios of B100 specimens at 8 weeks.

**Figure 4:**
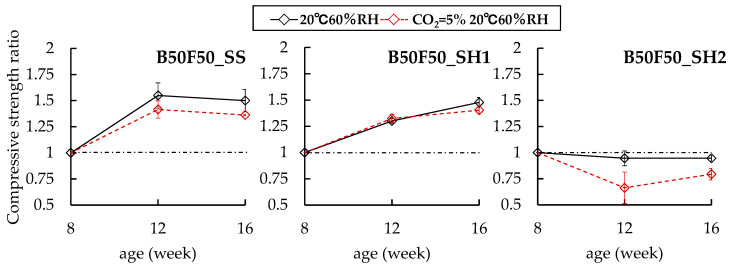
Changes in compressive strength ratios of B50F50 specimens at 8 weeks.

**Figure 5:**
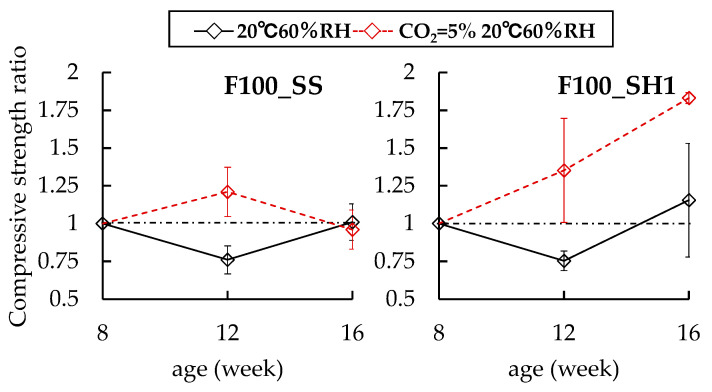
Changes in compressive strength ratios of F100 specimens at 8 weeks.

Figure 2 shows the compressive strength of each specimen at 8 weeks age. Figure 3, Figure 4 and Figure 5 show the changes in the compressive strength ratios with age for each specimen at 8 weeks age. First, in the B100 specimens, the strength at 8 weeks was 61.1 N/mm^2^ for B100_SS, 35.0 N/mm^2^ for B100_SH1, and 19.5 N/mm^2^ for B100_SH2. These measurements confirmed that the compressive strengths varied greatly depending on the type of alkali activator used. In the case of sodium silicate (SS) as the alkali activator, we considered that the compressive strength was different because the C–(N)–A–S–H structure was different from B100_SH1 and B100_SH2 because of the supply of Si from the alkali activator. Thus, it was confirmed that the effects of the type of alkali activator used on the compressive strengths were small in the B50F50 and F100 specimens. Further, it was confirmed that the strength of the F100 test piece was lower than those of the B100 and B50F50 test pieces. The B0_SH2 sample could not be demolded because of insufficient hardening at the age of 1 day. C.D. Atis et al. [29] investigated the effects of different curing temperatures on the compressive strengths using FA-based geopolymer mortars with different Na^+^ concentrations. Compared with their results, in the case of F100_SH2, we consider that the curing time and temperature were insufficient for the present method (60 °C, 6 h). 

In the case of the B100 specimens, it was confirmed that the change behaviors in the compressive strengths differed among the B100_SS, B100_SH1, and B100_SH2 samples in the accelerated carbonation environment. First, the B100_SS (CO_2_ = 0.03%) sample showed a compressive strength of 61.2 N/mm^2^ at 8 weeks of age and increased slightly until 12 and 16 weeks of age (compressive strength ratios of 1.03 at 12 weeks and 1.05 at 16 weeks). However, the compressive strength ratio of the cured in the accelerated carbonation environment decreased to 0.96 at 12 weeks (accelerated carbonation of 4 weeks) and 0.85 at 16 weeks (accelerated carbonation of 4 weeks); that is, differences in the compressive strengths were confirmed for the accelerated carbonation of the samples. Next, in the case of the B100_SH1 sample, the compressive strength ratio increased slightly for 8 weeks of age regardless of the accelerated carbonation environment (noncarbonated: 1.17 at 16 weeks, carbonated: 1.21 at 16 weeks). In the case of the B100_SH2 sample, which had a lower Na^+^ concentration than the B100_SH1 sample, the compressive strength ratio was 1.16 at 16 weeks (noncarbonated at 16 weeks). However, in the case of curing in the accelerated carbonation environment, the compressive strength ratio was 0.98 at 16 weeks (accelerated carbonation of 8 weeks), and a difference of 0.18 in the compressive strength ratio was confirmed based on the presence or absence of accelerated carbonation. With respect to such a result, F. Puertas et al. [22] described that in the case of using NaOH (Na_2_O/binder = 0.04) as the alkali activator, carbonation enhanced mortar cohesion possibly as a result of the precipitation of greater amounts of calcium carbonate in the pores, thereby causing a decline in the total porosity and average pore size and consequently an increase in the mechanical strength. In contrast, in the case of using sodium silicate (water glass) (Na_2_O/SiO_2_ = 0.85, Na_2_O/binder = 0.04) as the alkali activator, the decalcification of the C-S-H gel prompted by carbonation caused a loss of cohesion in the matrix and an increase in the porosity and decline in mechanical strength. It is generally known that in the carbonation of hardened cements using OPC, the pore structures become denser because of the formation of CaCO_3_, and the compressive strengths increase. Similarly, in the case of the B100_SS sample, which is rich in calcium, the compressive strength was expected to increase because of the formation of CaCO_3_ by carbonation; however, the compressive strength decreased. We speculate that the cause of this result is the collapse of the C–(N)–A–S–H structure and formation of shrinkage cracks due to decalcification of C–(N)–A–S–H; hence, it is necessary to study crack observations in the future. In the case of the B100_SH1 sample as well, it was estimated that the compressive strength decreased because of the change in the C–(N)–A–S–H structure; however, the compressive strength increased with age. We consider that this result may be related to factors such as the formation of carbonates. The results of XRD and ^29^Si MAS NMR are described in detail below.

In the case of the B50F50 specimens, B50F50_SS and B50F50_SH1 have increases in compressive strengths regardless of the presence of the accelerated carbonation environment. Further, no differences in compression strengths were observed due to accelerated carbonation. However, in the case of the B50F50_SH2 sample, which had a lower Na^+^ concentration than B50F50_SH1, it was confirmed that the compressive strength ratio decreased to 0.79 because of the accelerated carbonation. Finally, in the case of the F100_SS sample, no changes were observed in the compressive strength due to accelerated carbonation. However, in the case of the F100_SH1 sample, the compressive strength ratio of the accelerated carbonated specimen increased to 1.83 at 16 weeks. In the case of OPC, the pore structure becomes dense and the compressive strength increases because of the formation of CaCO_3_. Similarly, in the case of the F100_SH1 specimen, we concluded that the pore structure was densified because of the formation of carbonates, with a corresponding increase in the compressive strength. 

### 3.2. XRD

**Figure 6:**
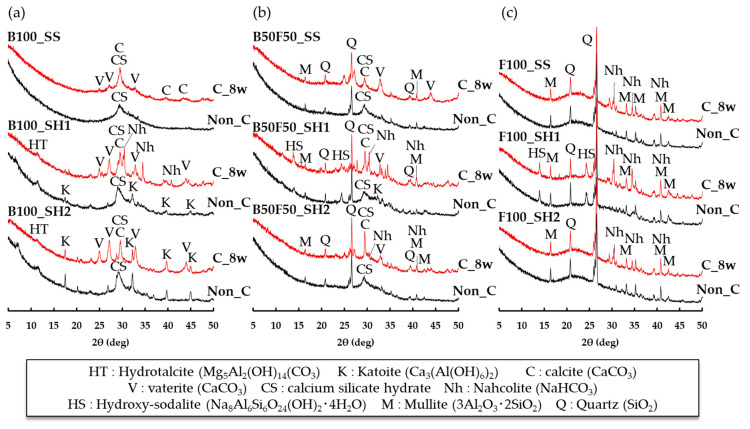
XRD results for paste specimens of (**a**) B100_SS, B100_SH1 and B100_SH2, (**b**) B50F50_SS, B50F50_SH1 and B50F50_SH2, and (**c**) F100_SS, F100_SH1 and F100_SH2, Non_C: 8 weeks, C_8w: 16 weeks (carbonation of 8 weeks).

Figure 6 shows the XRD measurement results of the paste specimens at 8 and 16 weeks (accelerated carbonation of 8 weeks). In the case of the B100_SS sample at 8 weeks, only the peaks of C–S–H (C–(N)–A–S–H) were confirmed; however, the peaks of hydrotalcite (Mg_5_Al_2_(OH)_14_(CO_3_), 2θ = 11.60°) [23,30] and katoite (Ca_3_(Al(OH)_6_)_2_, 2θ = 17.56°, 32.34°, 44.88°) were also confirmed in the B100_SH1 and B100_SH2 samples when using sodium hydroxide as the alkali activator. In addition, the XRD results of the B50F50 and F100 samples using fly ash confirmed peaks of mullite (3Al_2_O_3_·2SiO_2_, 2θ = 16.42°, 33.18°, 40.86°) and quartz (SiO_2_, 2θ deg 20.78°, 26.68°) in the FA. The formation of zeolite (called hydroxy-sodalite) (Na_8_Al_6_Si_6_O_24_ (OH)_2_·4H_2_O, 2θ = 14.04°, 24.52°) [31] was also confirmed in the B50F50_SH1 and F100_SH1 specimens with high Na^+^ concentrations. In the case of the B100_SS sample, the decrease in the C–S–H(C–(N)–A–S–H) peaks and increase in CaCO_3_ peaks of calcite (2θ = 29.6°, 39.5°, 43.6°,47.2°) and vaterite (2θ = 25.1°, 27.3°, 32.9°) were confirmed for accelerated carbonation. In the case of the OPC-based concrete, carbonation causes densification of the pore structure because of formation of CaCO_3_ composites, such as vaterite and calcite, thereby increasing the compressive strength. However, in the B100_SS sample, the strength decreased despite formation of CaCO_3_, as confirmed by the accelerated carbonation. We consider that shrinkage cracks occurred because of carbonation, resulting in the decrease in compressive strength. In addition, in the BFS_SH1 sample, the formation of Na-containing carbonate called nahcolite (NaHCO_3_) (2θ = 30.58°, 34.58°, 40.84°) was also confirmed. Therefore, we consider that Na^+^ is also carbonated in the case of AACs containing large amounts of Na^+^. In the case of the B50F50 specimens, it was confirmed that the C–S–H peaks decreased and that the CaCO_3_ peaks, such as those of calcite and vaterite, increased by accelerated carbonation regardless of the type of alkali activator used. Moreover, the peak of nahcolite (NaHCO_3_) was confirmed by accelerated carbonation in the B50F50_SH1 and B50F50_SH2 samples. In the F100 specimen, the formation of nahcolite was confirmed by carbonation; however, it was also confirmed that the change in the overall peak by carbonation was less than those in the B100 and B50F50 specimens. Therefore, in the case of F100, we consider that the change in the product by accelerated carbonation is small and that the formation of nahcolite is related to changes in the compressive strength because the formation of nahcolite was not confirmed in the B100_SS and B100_SH2 samples, whose compressive strengths decreased by accelerated carbonation. However, S.A. Bernal et al. [23] argue that natron (Na_2_CO_3_·10H_2_O) is most likely to be produced at atmospheric CO_2_ concentrations (0.03%) and that nahcolite (NaHCO_3_) is produced as the CO_2_ concentration increases. Therefore, it is necessary to study the reactions and effects at lower CO_2_ concentrations. 

### 3.3. TG/DTG Analyses

**Figure 7:**
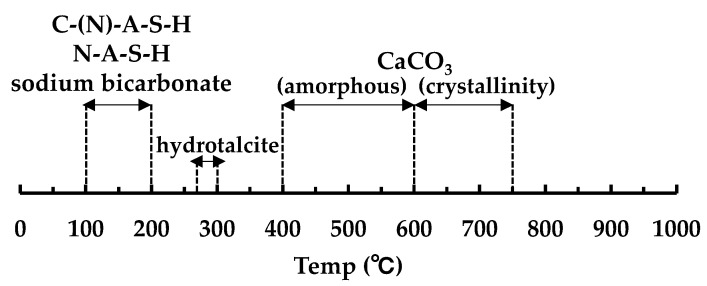
Decomposition temperature of hydrates and carbonates in TG/DTG.

**Figure 8:**
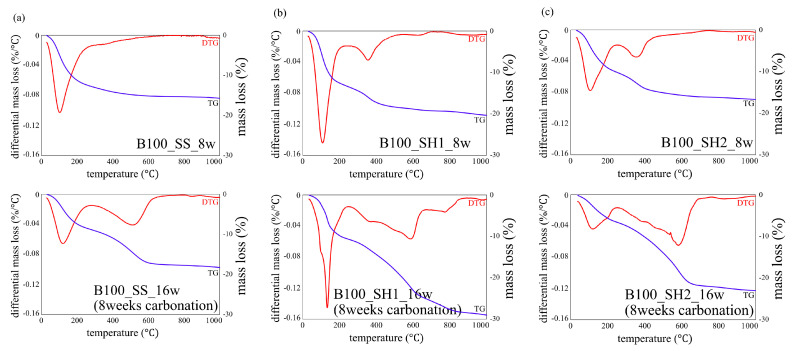
Thermogravimetric/differential thermal gravimetric (TG/DTG) analysis results for specimens of (**a**) B100_SS_8w and B100_SS_16w (8 weeks carbonation), (**b**) B100_SH1_8w and B100_SH1_16w (8 weeks carbonation), and (**c**) B100_SH2_8w and B100_SH2_16w (8 weeks carbonation).

**Figure 9:**
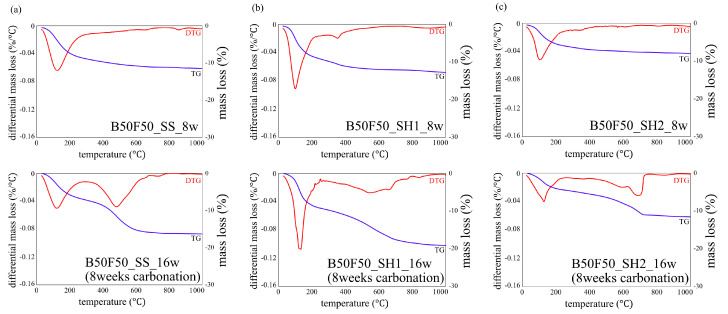
Thermogravimetric/differential thermal gravimetric (TG/DTG) analysis results for specimens of (**a**) B50F50_SS_8w and B50F50_SS_16w (8 weeks carbonation), (**b**) B50F50_SH1_8w and B50F50_SH1_16w (8 weeks carbonation), and (**c**) B50F50_SH2_8w and B50F50_SH2_16w (8 weeks carbonation).

**Figure 10:**
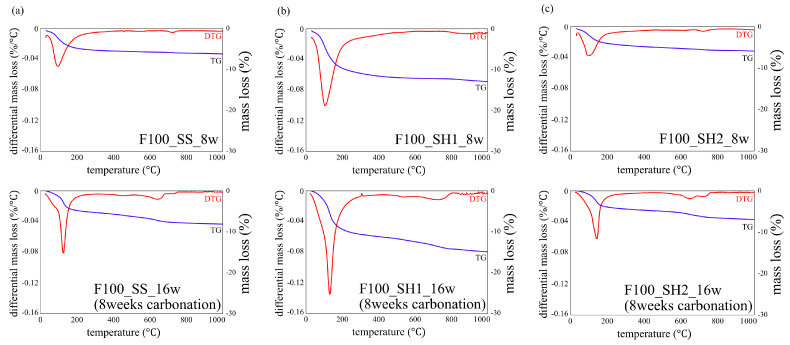
Thermogravimetric/differential thermal gravimetric (TG/DTG) analysis results for specimens of (**a**) F100_SS_8w and F100_SS_16w (8 weeks carbonation), (**b**) F100_SH1_8w and F100_SH1_16w (8 weeks carbonation), and (**c**) F100_SH2_8w and F100_SH2_16w (8 weeks carbonation).

Figure 7 shows the decomposition temperature of hydrates and carbonates in TG/DTG [32]. Figure 8, Figure 9 and Figure 10 show the TG/DTG results for accelerated carbonation of each specimen. First, in the results of the B100_SS_8w sample in Figure 8a, the decomposition of C–(N)–A–S–H gel is seen at 100–200 °C In the B100_SS_16w sample results, the amount of decomposition of the C–(N)–A–S–H gel decreased after accelerated carbonation, and the decomposition of amorphous CaCO_3_ was confirmed at 400–600 °C From these results, the process of carbonation by decalcification of the C–(N)–A–S–H gel was confirmed. In the B100_SH1 samples in Figure 8b, the decomposition of C–(N)–A–S–H at 100 to 200 °C as well as the decomposition of hydrotalcite at 270–300 °C are confirmed. While the decomposition of C–(N)–A–S–H gel due to carbonation is expected to decrease, the amount of decomposition does not change significantly; it is possible that in an environment with high Na^+^ concentration, the Na^+^ in the pore water is bound to the gel structure in the place of calcium eluted by carbonation in the C–(N)–A–S–H gel, thereby forming a gel structure rich in Na^+^. From this, a difference in the gel structure after carbonation is expected based on the Na^+^ concentration. In addition, there is the possibility that sodium bicarbonate or sodium carbonate generated by carbonation of excess Na^+^ in the pore water may cause such effects, as confirmed by the XRD results in the next section. From the changes based on the accelerated carbonation of the B100_SH1 sample, amorphous CaCO_3_ at 400–600 °C as well as decomposition of CaCO_3_ with high crystallinity at ≈ 750 °C are confirmed. Accordingly, under the accelerated carbonation conditions, different carbonation mechanisms are identified depending on the type of activator used. In the low Na^+^ concentration of the B100_SH2 sample in Figure 8c, decomposition is observed at the same temperature as the B100_SH1 sample, but there is a difference in the amount of decomposition. In addition, unlike the B100_SH1 specimen, which has high Na^+^ concentration, the amount of decomposition at 100–300 °C decreases owing to carbonation, and CaCO_3_ formation with high crystallinity cannot be confirmed. From this result, it can be concluded that the Na^+^ concentration or the pH in the pore solution may affect the crystallinity of CaCO_3_. 

In the B50F50_SS and B50F50_SH1 samples shown in Figure 8b and Figure 9a, there are differences in the degree, but similar tendencies as those of the B100 specimen are confirmed. However, in the B50F50_SH2 sample, unlike the B100_SH2 specimen, the decomposition of CaCO_3_ with high crystallinity was observed. In the case of the BFS/FA blend, it is known that C–(N)–A–S–H and N–A–S–H gels are formed simultaneously; hence, it can be assumed that not only the Na^+^ concentration but also the structure of the gel formed due to the type of binder would affect the crystallinity of the CaCO_3_ produced under accelerated carbonation conditions. From the results of the F100 sample shown in Figure 10, the difference between the decomposition amounts of 100–200 °C at high and low levels of Na^+^ concentration is confirmed [32]; this difference is considered to depend on the degree of formation of the N–A–S–H gel. In addition, the decomposition from sodium bicarbonate or sodium carbonate is slightly increased owing to the overall carbonation level, and this increase is remarkable in specimens with high Na^+^ concentrations.

### 3.4. ^29^Si MAS NMR

**Figure 11:**
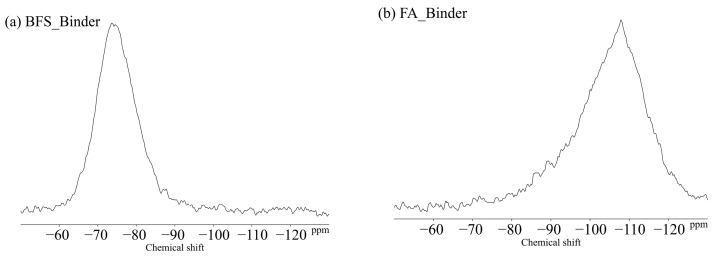
Solid-state ^29^Si MAS NMR chemical shifts for (**a**) BFS and (**b**) FA-binders.

**Figure 12:**
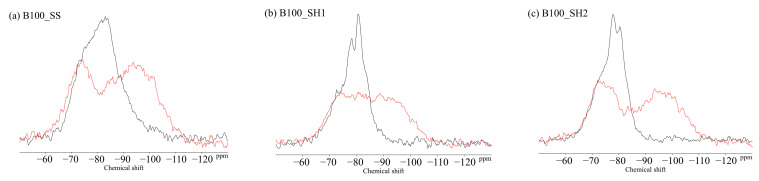
Solid-state ^29^Si MAS NMR chemical shifts for (**a**) B100_SS, (**b**) B100_SH1, and (**c**) B100_SH2 samples.

**Figure 13:**
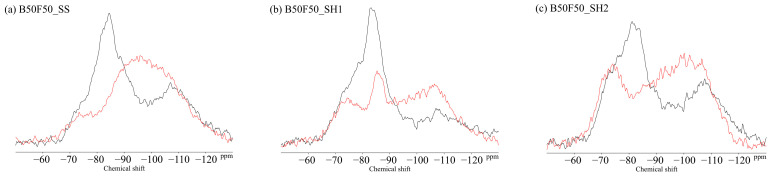
Solid-state ^29^Si MAS NMR chemical shifts for (**a**) B50F50_SS, (**b**) B50F50_SH1, and (**c**) B50F50_SH2 samples.

**Figure 14:**
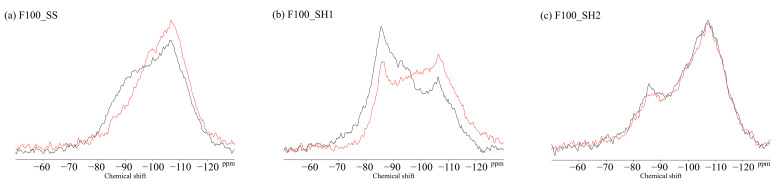
Solid-state ^29^Si MAS NMR chemical shifts for (**a**) F100_SS, (**b**) F100_SH1, and (**c**) F100_SH2 samples.

Figure 11 shows the ^29^Si MAS NMR results for the BFS and FA binders used in this study. The unreacted silicates appeared mainly in Q^0^ and Q^1^ for the BFS and Q^4^ for the FA. Figure 12 shows the changes due to accelerated carbonation of the B100 specimens. First, at the age of 8 weeks (black line), the formation of the C–(N)–A–S–H structures were observed in peaks Q^1^ to Q^2^ regardless of the type of activator. Moreover, decalcification occurs under accelerated carbonation for 8 weeks, and it is confirmed that the C–(N)–A–S–H gel structure is altered. Here, in the case of the B100_SH1 sample with high Na^+^ concentration, the peaks at −80 to −90 ppm after carbonation are relatively stronger compared to the two samples at low Na^+^ concentration. This is related to the aforementioned TG/DTG results, and it is expected that the Na^+^ is bound in a gel structure instead of the Ca to be carbonated. From the results of the compressive strength tests, in the case of the B100_SS specimen, the compressive strength is observed to decrease with carbonation, which is expected to be caused by cracking from carbonation shrinkage because of the changes to the gel structure. However, in the B100_SH1 sample, similar to the B100_SS specimen, the gel structure collapse was evident in the ^29^Si NMR spectra, but the compressive strength did not decrease; moreover, it is expected that in the high Na^+^ concentration environment, the Na^+^ is bound to the gel structure, resulting in relatively small shrinkage. Accordingly, in the future, it is necessary to examine the amount of shrinkage based on the type of activator used and the Na^+^ concentration. The results of the B50F50 samples in Figure 13 show the different binding states depending on the Na^+^ concentrations, but these are not related to the type of activator used. In the B50F50_SS and B50F50_SH2 specimens, where the Na^+^ concentration is relatively low, the Q^4^ peaks appear to be relatively large in addition to the Q^0^–Q^1^ peaks, which is attributed to the unreacted FA owing to the lack of Na^+^. In the B50F50_SH1 sample where the Na^+^ concentration is high, the peak corresponding to the unreacted FA decreases, and the formation of the C–(N)–A–S–H gel in the Q^1^–Q^2^ range is confirmed. The carbonation trends of the B50F50 samples are also similar to those of the B100 samples. In the results for the F100 samples in Figure 14, the degree of polymerization of the gel formed owing to the Na^+^ concentration is observed to have differences. For the F100_SS and F100_SH2 specimens with low Na^+^ concentrations, the N-A-S-H formation was centered around the Q^4^ peak, and no significant changes were observed in this peak from carbonation. For the F100_SH1 sample, it was confirmed that gel peaks centered on Q^1^ and Q^2^ were formed, and the N-A-S-H structure changed slightly from carbonation.

### 3.5. pH and Neutralization Depth

**Figure 15:**
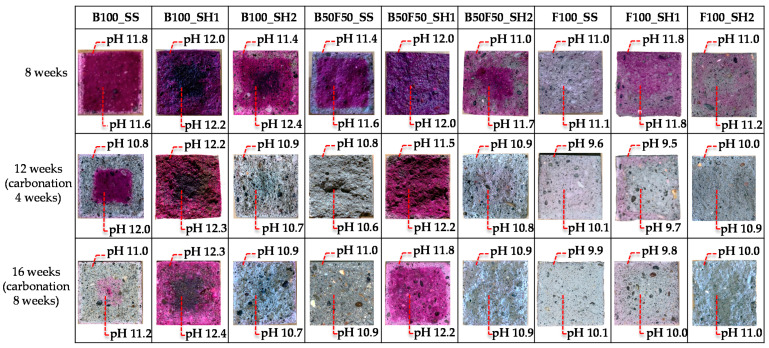
Changes in the colored areas of the mortar specimens after spraying with phenolphthalein and the corresponding changes in the pH values.

Figure 15 shows the changes in the color of the mortar specimens after spraying with phenolphthalein at 8 weeks of age as well as 4 and 8 weeks of accelerated carbonation. In addition, the results of the pH outside the cross section (5 mm from the surface) and at the center of the section (20 mm square at the center) are shown. The pH of the specimen affects the stability of the hydrate, because C–(N)–A–S–H is stable at high pH (>12) and N-A-S-H is stable at low pH (<12) [33].

First, from the results for the normal samples at 8 weeks, it is confirmed that the F100_SS specimen did not change color from the phenolphthalein solution compared to the other specimens. Furthermore, it is confirmed that the pH was 11.0 outside the chosen area and 11.1 at the center of the section, which was lower than the other measured values. Moreover, in the case of the F100_SH2 sample, the color change because of the phenolphthalein solution was slightly confirmed; however, the pH was as low as 11.0 outside the area of interest and 11.2 at the center of the section. In the case of hardened cement using OPC, the pore solution maintains a high pH mainly because of the presence of calcium hydroxide (Ca(OH)_2_). However, for the AACs, the Na^+^ (or K^+^, Li^+^) contained in the alkali activator are dissolved in the pore solution to maintain the pH. Further, FA-based AACs are considered to have low pH values because large amounts of Na^+^ are consumed in the production of N-A-S-H, and the amount of Na^+^ dissolved in the pores is small. In the case of the F100_SH1 sample, the color change due to the phenolphthalein solution was confirmed, and the pH result was 11.8, which was higher than that of 11.0 for the F100_SS specimen; this may be attributed to the different amounts of Na_2_O in the alkali activators. In the cases where SS and SH2 were used as the alkali activators, Na_2_O/binder = 0.045, whereas for SH1, Na_2_O/binder = 0.123, with the amount of Na_2_O being higher than those of SS and SH2. Therefore, we concluded that the amount of Na dissolved in the pore solution also increased and that such differences occurred between the F100_SH1 and F100_SS or F100_SH2 samples. 

Next, from the changes in each specimen for the accelerated carbonation cases, with the exception of the B100_SH1 specimen, decrease in the color range and decrease in the pH were confirmed. However, in the case of the B100_SH1 sample, the color range and pH did not change by accelerated carbonation; herein, we considered that although carbonation progressed in the case of the B100_SH1 sample, the pH did not change because of the formation of highly soluble carbonate in the accelerated carbonation process. In addition, the B100_SH2 and B100_SS samples show regions with color changes and different pH owing to accelerated carbonation. This decrease in pH was attributed to the decrease in the Na^+^ concentrations in the pore solutions, which was assumed to be because of decrease of Na^+^ concentration in the pore solution from adsorbing Na^+^ to the decalcified C–(N)–A–S–H. The results of the ^29^Si MAS NMR show that the C–(N)–A–S–H structure is polymerized because of the chemical shift to the Q^3^ and Q^4^ sides. We consider that C–(N)–A–S–H and N–A–S–H, which are structurally different from C–(N)–A–S–H before carbonation, are produced by carbonation because Ca^2+^ is eluted by decalcification of C–(N)–A–S–H, and the Na^+^ and Al^3+^, which have lower binding strengths than Ca^2+^, are bound to Si. J. Kim et al. [21] suggested that in the case of carbonation of cement paste with sodium silicate, the structure is similar to N–A–S–H or (C, N)–A–S–H, which have free structures compared to ordinary C–S–H because of the binding of Si^4+^, Al^3+^, and Na^+^ by decalcification. In this study, we consider that the pH decreased owing to the decrease in Na^+^ concentration in the pore solution because the Na^+^ binds to Al^3+^ and Si^4+^ after decalcification of C–(N)–A–S–H by carbonation. In addition, the comparison of each binder confirmed that the pH decreased in the order of B100, B50F50, and F100. As mentioned above, we attribute this to the amount of Na^+^ bound as N-A-S-H. Thus, it was confirmed via comparisons of the alkali activators that the pH values of the specimens were relatively high when using SH1. This result is the same as that noted above, and is assumed to be caused by the amount of Na_2_O in the alkali activator, which was higher in SH1 than in SS and SH2, thus causing more Na^+^ to be dissolved in the pore solution.

## 4. Discussion on Compressive Strength Drop under High CO_2_ Conditions

In the results of the compressive strength, significant decrease in compressive strength was observed in the specimens using SS as the BFS base. At the level of SH2 addition, it is thought that the strength-generating structure was not formed due to insufficient alkali activator. Regarding this decrease in compressive strength, we predicted that carbonation shrinkage caused by structural changes of C–(N)–A–S–H under high-concentration CO_2_ conditions was the cause. Therefore, we observed changes in pore structure due to high concentration accelerated carbonation on the B100_SS test specimen, which showed a decrease in compressive strength. As a comparison group for the experiment, B100_SH with no decrease in strength at the level based on BFS, and B50F50_SS specimens containing BFS and SS but with no decrease in strength were used. Mercury intrusion porosimetry (MIP) was performed using a Quantachrome/PoreMaster33. The measurement samples were prepared by mortar specimen into diameter 5 × height 5 mm size cylinders, performing vacuum freeze-drying for 24 h for thorough drying [34].

**Figure 16:**
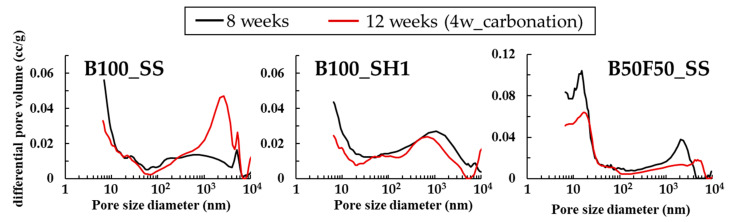
Pore structure analysis by MIP of 8 weeks, 12 weeks (accelerated carbonation 4 weeks).

Figure 16 shows the results of pore structure analysis by MIP of mortar specimens of 8 weeks, 12 weeks (accelerated carbonation 4 weeks) are shown. In B100_SS, whose compressive strength decreased by carbonation, it was predicted that the pores would become coarser due to changes in the C–(N)–A–S–H structure, and the expected result was confirmed. The pores around 10 nm are slightly reduced, but the pores around 100–4000 nm are relatively largely increasing, which may cause a decrease in compressive strength. On the other hand, as a result of B100_SH1, pore densification in the range of 100–10,000 nm by carbonation is confirmed. In the XRD results, the formation of carbonates containing Na^+^ was confirmed by carbonation, which is considered densification by the formation of carbonates. On the other hand, in B50F50_SS, which does not decrease in strength by carbonation, the pore structures at around 10 nm and 100–4000 nm are densified by carbonation. The effect of carbonation of C–(N)–A–S–H is considered to be small, because B50F50_SS has a lower Ca / Si ratio than B100_SS. 

**Figure 17:**
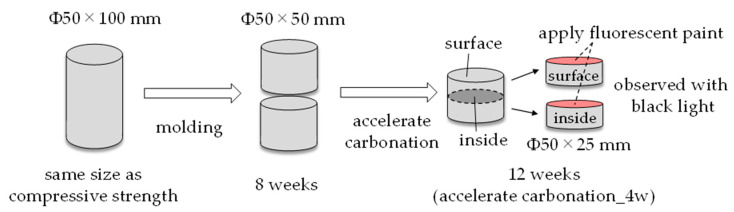
Method of the crack observation.

For crack observation, φ50 × 100 mm, which is the same size as the compressive strength, was used. The test piece was formed into φ50 × 50 mm at 8 weeks of age, and then accelerated carbonation was performed. At 12 weeks of age (4 weeks of accelerated carbonation), the specimen was molded from φ50 × 50 mm to φ50 × 25 mm in order to observe both the surface and the inside of the specimen. After that, a fluorescent paint was applied to the cross section of the test piece, and cracks were observed using black light to make it easier to see the cracks (Figure 17). 

**Figure 18:**
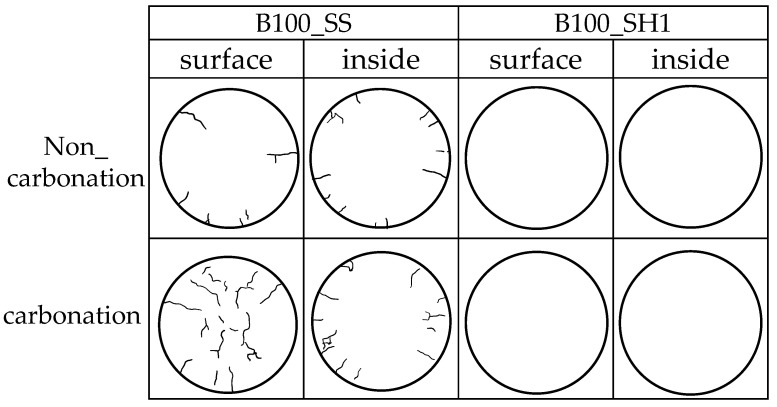
Crack observation of 12 weeks (accelerated carbonation 4 weeks).

Figure 18 traced only the cracks to make the cracks easier to see. In B100_SS, it was confirmed that accelerated carbonation caused cracks in the entire specimen. The occurrence of this crack is considered to be the direct cause of the decrease in compressive strength by carbonation. From this result, in the accelerated carbonation behavior of the test specimen including BFS and SS, decalcification of the C–(N)–A–S–H structure lowers the cohesive force of the entire structure, and cracks can be generated from the change of the pores. It was confirmed that cracking occurred even without accelerating carbonation, which may be affected by the relatively fast curing speed of fresh mortar using BFS and SS. Here, in the cracking of B100_SS (specimens using only BFS and sodium silicate) in an environment without accelerated carbonation (actual environment), it is unclear whether a decrease in compressive strength may occur from a long-term perspective. On the other hand, no cracks were confirmed in B100_SH1. Here, from the results of XRD, TG-DTG, and ^29^Si MAS NMR using the paste specimens, decalcification of C–(N)–A–S–H that appears in B100_SS is confirmed in B100_SH1 as well. However, considering the matrix of the mortar specimen, carbonation occurs only on the surface of the specimen. The formation of carbonates containing Na^+^ on the surface helps to suppress the penetration of CO_2_. 

## 5. Conclusions

In this study, we examined the changes in the physical and chemical properties of AACs before and after carbonation using different binders and alkali activators. The results from this study can be summarized as follows:

1. In the case of specimens using BFS and sodium silicate, the compressive strength is reduced by accelerated carbonation. This is based on the change in the pore structure of the specimen and the occurrence of cracks.
2. In the case of specimens using BFS and sodium silicate, even in the environment without accelerated carbonation, some cracks were observed, but the compressive strength did not decrease. It is necessary to additionally observe the decrease in compressive strength in the long-term view of the formulation using only BFS and sodium silicate.
3. In the case of the BFS and BFS with FA specimens, we confirmed that accelerated carbonation reduced the XRD peaks and amount of C–(N)–A–S–H produced by generating amorphous CaCO_3_ vaterite in addition to calcite. Further, we confirmed that nahcolite was produced at high Na^+^ concentrations.
4. In the case of BFS and BFS with FA specimens, changes in the C–(N)–A–S–H structures were confirmed regardless of the type of alkali activator used. However, for FA specimens, the change in the chemical shift due to accelerated carbonation was small; hence, the change in the N-A-S-H structure was also considered to be small.
5. B100_SH suppresses the permeation of CO_2_ by the formation of carbonate containing Na^+^, but considering that the solubility of carbonates containing Na^+^ is considerably higher than that of CaCO_3_, we need to perform exposure tests under real conditions with a water supply.

## Figures and Tables

**Table 1:** Chemical Compositions (wt.%) and Physical Properties of bfs and fa.

Type	Surface Area (cm^2^/g)	Density (g/cm^3^)	Chemical Composition (Mass%)					
			CaO	SiO_2_	Al_2_O_3_	Fe_2_O_3_	MgO	SO_3_
BFS	3930	2.91	43.36	34.03	14.36	0.83	6.51	-
FA	4010	2.29	3.75	57.75	23.65	5.75	1.1	1.05

**Table 2:** Preparation of Mortar Specimens (g/L).

Index	Water/Binder	Binder:Sand	Binder		Alkali Activator		Water	Sand	Na_2_O/Binder
			BFS	FA	WG	NaOH			
B100_SS	0.4	1:2	669	0	167	0	192	1338	0.045
B100_SH1			665	0	0	106	242	1330	0.123
B100_SH2			667	0	0	38	258	1334	0.045
B50F50_SS			322	322	163	0	186	1300	0.045
B50F50_SH1			324	324	0	103	235	1292	0.123
B50F50_SH2			324	324	0	37	252	1296	0.045
F100_SS			0	625	158	0	180	1260	0.045
F100_SH1			0	630	0	99	228	1251	0.123
F100_SH2			0	629	0	36	243	1258	0.045

B: BFS, F: FA, SS: sodium silicate, SH: sodium hydroxide

**Table 3:** Preparation of Paste Specimens (g/L).

Index	Water/Binder	Binder		Alkali Activator		Water	Na_2_O/Binder
		BFS	FA	WG	NaOH		
B100_SS	0.4	1369	0	342	0	392	0.045
B100_SH1		1349	0	0	215	491	0.123
B100_SH2		1346	0	0	77	522	0.045
B50F50_SS		644	644	322	0	368	0.045
B50F50_SH1		635	635	0	202	462	0.123
B50F50_SH2		633	633	0	73	491	0.045
F100_SS		0	1214	304	0	348	0.045
F100_SH1		0	1198	0	191	436	0.123
F100_SH2		0	1196	0	67	465	0.045

B: BFS, F: FA, SS: sodium silicate, SH: sodium hydroxide

**Table 4:** Measurement Items.

Type	Mortar Specimen		Paste Specimen
Size	φ50 × 100 mm	40 × 40 × 160 mm^3^	Powder
measurement	Compressive strength	pH and neutralization depths	XRD TG-DTG ^29^Si MAS NMR

## Data Availability

Data available on request due to restrictions eg privacy or ethical.
